# Supplementary material for: Assessing the risk of Guillain-Barré syndrome in older adults after bivalent RSV pre-F vaccination in England
Source: Nat Commun. 2025 Dec 11;16:11496. doi: 10.1038/s41467-025-66280-z (PMC12748555; doi:10.1038/s41467-025-66280-z)
Supplement: Supplementary file 1 — Supplementary Information [file 41467_2025_66280_MOESM1_ESM.pdf]

Supplementary: Calculation of attributable risk adjusting for differential positive predictive value as seen in Lloyd et al.

In the study by Lloyd at el [1] they report a differential positive predictive values (PPV) for cases in the risk (PPV=62%) and control windows (PPV=82%) following bivalent pre-F RSV vaccine.

If we apply these positive predictive values to our study then the number cases in the risk window would reduce to 0.62x48 = 29.8 and in the control window to 0.82x35 = 28.7

With 48 cases in the risk window and 35 in the control window (a ratio of 48/35=1.37) we observed a relative incidence (RI) of 3.34.

With a new ratio of 29.8 / 28.7 = 1.04 cases in the risk to control windows this would be expected to give a lower RI of about 3.34 x (1.04/1.37) = 2.53.

With a RI of 2.53 and with 29.8 cases in the risk window this gives an estimated attributable number of cases of 29.8 x (1.53/2.53) = 18.0.

Based on 1,483,800 doses given this gives an AR of 12.1 per million.

Reference

[1] Lloyd PC, Shah PB, Zhang HT, Shah N, Nair N, Wan Z, et al. Evaluation of Guillain-Barré syndrome following Respiratory Syncytial Virus Vaccination among Medicare Beneficiaries 65 Years and Older. medRxiv. 2025:2024.12.27.24319702.
